# The Genome of an Ocean Wanderer: A High‐Quality Long‐Read Reference Genome for the Trindade Petrel (*Pterodroma arminjoniana*)

**DOI:** 10.1002/ece3.74568

**Published:** 2026-10-21

**Authors:** Mariana Scain Mazzochi, Olga L. Herrera‐Trujillo, Ken Norris, Malcolm A. C. Nicoll, Paula Plaza, David A. Stewart, Maria P. Dias, Mónica C. Silva, Leandro Bugoni

**Affiliations:** ^1^ Universidade Federal do Rio Grande—FURG, Instituto de Ciências Biológicas Rio Grande RS Brazil; ^2^ Department of Animal Biology, Faculty of Sciences of the University of Lisbon CE3C—Centre for Ecology, Evolution and Environmental Changes & CHANGE —Global Change and Sustainability Institute Lisboa Portugal; ^3^ Natural History Museum at Tring Hertfordshire UK; ^4^ Institute of Zoology Zoological Society of London London UK; ^5^ Center of Ecology and Sustainable Management of Oceanic Island (ESMOI), Facultad de Ciencias del Mar Universidad Católica del Norte Coquimbo Chile; ^6^ Instituto de Ecología y Biodiversidad (IEB) Santiago Chile; ^7^ Department of the Environment, Tourism, Science and Innovation Queensland Government Brisbane Queensland Australia

**Keywords:** allochrony, hybridisation, long‐read, Procellariiformes, proteome

## Abstract

The Trindade petrel ( *Pterodroma arminjoniana* ) is a procellariiform seabird found on Trindade Island in the South Atlantic Ocean. Recent evidence suggests that it also ranges and breeds across the Western Indian and South Pacific Oceans, where it hybridises with closely related species. The fitness effects of hybridisation, and the extent to which these might vary among populations, have yet to be studied in detail. Some populations contain seasonally distinct breeding sub‐populations, but the causes and consequences of this variation are unknown. While whole‐genome sequencing (WGS) is revolutionising our understanding of eco‐evolutionary processes such as hybridisation and local adaptation, there is a lack of high‐quality reference genomes in the Trindade petrel clade. Until recently, whole‐genome assemblies in *Pterodroma* petrels were limited to a small number of species and were largely based on short‐read sequencing. Oxford Nanopore Technology (ONT) is enabling long‐read sequencing of non‐model species, and this approach has recently been used to generate a high‐quality reference genome for *Pterodroma*. Here, we use ONT to produce a high‐quality long‐read reference genome for *P. arminjoniana* . We first generated a *de novo* assembly that we then scaffolded using the reference genome of a closely related species, the Cory's shearwater (*Calonectris borealis*). The resulting assembly, generated from a male bird, was 1.25 Gbp in size, had a scaffold N50 of 85 Mbp, and a BUSCO completeness score of 97.6%. Our annotation identified 18,099 protein‐coding genes, of which 92.1% corresponded to lineage‐consistent avian gene families, and no contamination was detected. We also compared the new reference with other *Pterodroma* genomes using a multispecies‐coalescent phylogenomic analysis. Our *P. arminjoniana* reference genome will be a valuable resource for ecological and evolutionary studies of this species and its close relatives, as well as for comparative genomic research on Procellariiformes more generally.

## Graphical abstract

We present the first whole‐genome assembly for the Trindade petrel ( *Pterodroma arminjoniana* ), a wide‐ranging procellariiform seabird of conservation and eco‐evolutionary importance. These resources provide a reference‐quality genome for future studies of taxonomy and systematics, morphology, hybridisation, gene flow, population structure, conservation genomics, and comparative genomics in Trindade petrels and related gadfly (Pterodroma) petrels.

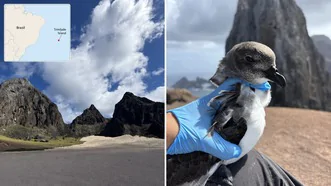

## 1 Introduction

The avian order Procellariiformes, which includes albatrosses, petrels, shearwaters and storm petrels, is the great ocean wanderers. Individuals of these species spend a considerable amount of time at sea where they can cover extraordinary distances on foraging trips both during their breeding season and non‐breeding migrations (Jouventin and Weimerskirch 1990; Phillips et al. 2007; Franklin et al. 2022; Beal et al. 2021; Ramírez‐Bal et al. 2026). They typically breed on isolated, predator‐free islands every 1–2 years (Schreiber and Burger 2001). Where closely related species co‐occur at their nesting sites, hybridisation is often recorded (Booth‐Jones et al. 2017; Brown et al. 2015; Phillips et al. 2018; Izquierdo‐Aranega et al. 2025; Plaza et al. 2023). On some islands, there is temporal segregation of breeding species (allochrony) (Ramos et al. 2016), which interestingly can also occur within a species with sub‐populations breeding on the same island but at different times. This can ultimately lead to temporal divergence and sympatric speciation (Friesen et al. 2007).

Both hybridisation and temporally distinct breeding sub‐populations occur in the Trindade petrel ( *Pterodroma arminjoniana* ). This species originates from Trindade Island in the South Atlantic Ocean, but genetic and tracking data suggest it ranges widely in the Western Indian and Southern Pacific Oceans (Booth‐Jones et al. 2017; Franklin et al. 2022). It regularly hybridises with other closely related *Pterodroma* species across this range, particularly *P. neglecta* and *P. heraldica* (Booth‐Jones et al. 2017). Interestingly, it has colonised Round Island off the north coast of Mauritius (W Indian Ocean) over the last century, where it co‐occurs and hybridises with several other closely related Pacific *Pterodroma* species (Booth‐Jones et al. 2017; Brown et al. 2010). On Trindade Island, there is evidence of distinct sub‐populations breeding at different times (Fonseca‐Neto 2004), which may cause population structure (Hendry and Day 2005), or reflect different patterns of gene expression (Laine et al. 2019).

These patterns raise some interesting eco‐evolutionary questions. Does hybridisation have a positive or negative effect on the fitness of the hybrids? Do these fitness effects vary between islands or contributing parental species? Is temporal variation in breeding seasonality within populations leading to genetic (or transcriptomic) differentiation? Might this temporal segregation ultimately lead to sympatric speciation? Historically, hybridisation in birds has been considered to largely have negative fitness effects, but it can also have positive effects through the rapid generation of adaptive genotypes/phenotypes, or through genetic rescue in species that have lost significant genetic diversity (Izquierdo‐Aranega et al. 2025; Todesco et al. 2016; Horta et al. 2025). Temporal variation in breeding seasonality within populations has likely driven sympatric speciation in storm petrels (Friesen et al. 2007), raising the possibility that similar processes might be operating in Trindade petrels.

Whole genome sequencing (WGS) is revolutionising our ability to address such questions (Taylor and Larson 2019; Horta et al. 2025; Jorquera et al. 2025). Despite this potential, applying these approaches to Trindade petrels and their close relatives has been limited by the availability and contiguity of suitable reference genomes. Recently, a high‐quality reference genome was produced for the Galápagos petrel ( *Pterodroma phaeopygia* ) (Sessi et al. 2026), and genomes based on short‐read sequencing are beginning to emerge from the Birds 10 K (B10K) consortium (https://b10k.com), including for *P. arminjoniana* . Nevertheless, a highly contiguous, high‐quality reference genome for Trindade petrels will provide significant genomic resources for downstream analyses exploring morphological variation (e.g., colour polymorphism), hybridisation, gene flow, allochrony and population structure, as well as supporting fundamental studies in taxonomy and systematics. Genomic data also have conservation value (Hogg 2024), including, for example, assessing species at risk, and informing management strategies. This is particularly true for highly threatened species such as those in the *Pterodroma* genus, which have suffered massive population declines in the past and are still at risk due to threats on land and at sea (Rodríguez et al. 2019; Dias et al. 2019).

Here, we describe the production of a high‐quality long‐read reference genome for the Trindade petrel. We used Oxford Nanopore Technology (ONT), which is increasingly enabling genome sequencing of non‐model organisms in their country of origin (Sessi et al. 2026). This approach substantially reduces the time and infrastructure required for the generation of genomic libraries and enables the sequencing of long reads, which is crucial for assembling a reference‐quality genome.

## 2 Methods

### 2.1 Study Species and Study Area

The Trindade Petrel breeds on Trindade Island, Atlantic Ocean, and on Round Island (near Mauritius), Indian Ocean (Brown et al. 2010). Trindade Island is located about 1200 km off the Brazilian coast (Figure 1), and its extensive deforestation, largely driven by fires and introduced goats, is considered responsible for the extinction of some native species, such as the red‐footed Booby *Sula sula* (Muraro et al. 2025). Here, the petrels nest on rocky substrates, primarily in cavities within the volcanic outcrops surrounding the crater of the extinct volcano at Praia das Tartarugas (Figure 1), Praia do Lixo (Ilha do Sul), and Pico do Monumento. There are two temporally distinct breeding seasons of Trindade petrels (Fonseca‐Neto 2004), and both sub‐populations use the same nesting habitat. The species is classified as Vulnerable globally, but Critically Endangered in Brazil (MMA 2022). The introduction of house mice ( *Mus musculus* ) remains an ongoing threat (Alves and Aguirre‐Muñoz 2011).

**FIGURE 1:**
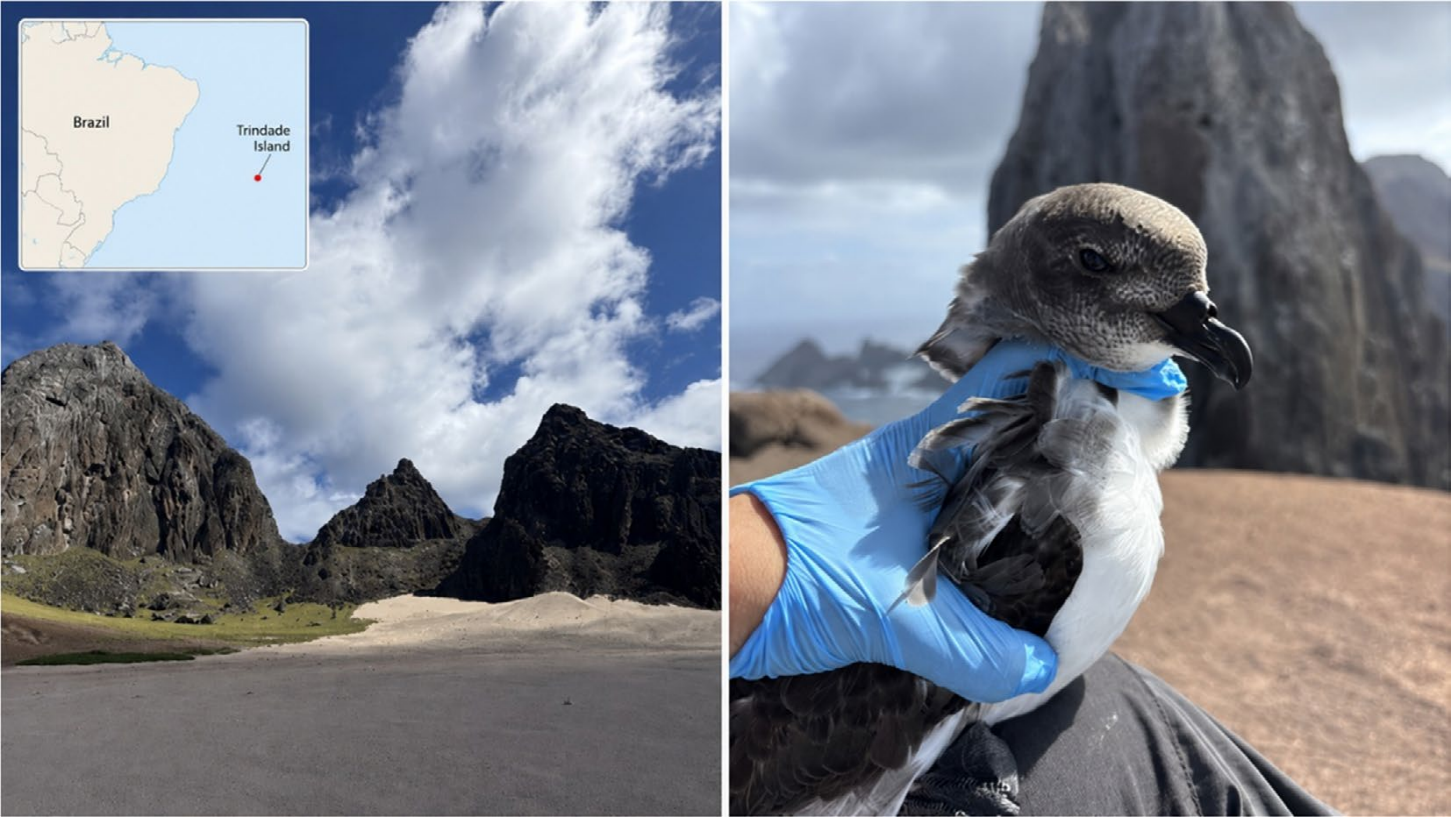
Study area and study species. The map inset shows the location of Trindade Island in the South Atlantic Ocean. The left panel shows one of the main nesting areas at Praia das Tartarugas. The right panel shows a pale morph Trindade petrel being handled for ringing and sampling (this is not the individual that was sampled for the genome).

The species occurs as light, intermediate and dark morphs; on Round Island, the two latter morphotypes are the most frequent, whereas on Trindade, the light morph is the most common (Leal et al. 2019). Birds mate assortatively by plumage type (Leal et al. 2019), and lay a single egg per breeding season with biparental care of the chick (Fonseca‐Neto 2004). During the breeding season, the distribution at sea of the population from Trindade includes the southwestern Atlantic Ocean, and a broad area east of Trindade Island; during the non‐breeding season, the birds use the North Atlantic Subtropical Gyre (Leal and Bugoni 2021).

### 2.2 Field Sampling

One adult Trindade petrel breeding on Trindade Island was captured using a dip net and banded with a CEMAVE ring (N37024) in April 2022 at Praia das Tartarugas. The same bird was recaptured in May 2024 and approximately 500 μL of blood was collected from the brachial vein, stored in absolute ethanol, and subsequently stored at –20°C at the scientific station on Trindade Island. Logistical constraints associated with transport from the island resulted in the sample being stored in an ultralow‐temperature freezer (−80°C) 1 week after collection.

### 2.3 DNA Extraction and Sequencing

The sample was centrifuged to pellet the blood components, and the ethanol was removed. Cells were washed with 1× PBS to remove residual ethanol. Genomic DNA was extracted using the Monarch HMW DNA Extraction Kit for Tissue, following the manufacturer's recommendations. This kit was selected based on the protocol tested and recommended by Oxford Nanopore Technologies for the Ultra‐Long DNA Sequencing Kit V14 (SQK‐ULK114). DNA concentration was measured using Qubit Fluorometric Quantitation (Thermo Fisher Scientific) with the Qubit 1X dsDNA High Sensitivity Assay Kit.

To maximise genome coverage and optimise the use of limited sample material, three types of libraries were constructed from the sampled individual. One library was prepared using the Ultra‐Long DNA Sequencing Kit (SQK‐ULK114; Oxford Nanopore Technologies). For DNA extractions that yielded insufficient DNA concentrations for the ultra‐long protocol, two additional libraries were constructed using the Rapid Sequencing DNA Kit (SQK‐RBK114‐96) and the Ligation Sequencing gDNA Kit (SQK‐NBD114‐96). In these instances, the sample was multiplexed across at least four barcodes, following the manufacturer's instructions.

Sequencing was performed on FLO‐PRO114M flow cells using a PromethION 2 Solo sequencer (Oxford Nanopore Technologies) connected to a Linux workstation using molecular facilities at the Subtropical Biodiversity Center, Universidade Federal do Rio Grande (CBS‐FURG). To maximise pore utilisation, six new and three previously used flow cells were employed. The sequencing process was monitored in real time using the MinKNOW software (Oxford Nanopore Technologies), which was configured to perform rapid basecalling during the run. Raw sequencing signals were stored locally as POD5 files and subsequently processed using the super accuracy mode for basecalling. Details of the data generated by each library are given in Table 1.

**TABLE 1:** Sequencing yield and read‐quality metrics for the three Oxford Nanopore library preparations used to generate the *P. arminjoniana* genome.

Library	Reads	Yield (Gbp and %age)	Coverage	Mean read length (bp)	Read N50 (bp)	Mean read quality	Reads ≥ Q20 (%)
SQK‐NBD114‐96	1,737,836	7.39 (13.0%)	5.9x	4252	11,788	18.4	61.3
SQK‐RBK114‐96	322,039	1.60 (2.8%)	1.3x	4964	11,998	14.6	42.7
SQK‐ULK114	4,661,010	48.03 (84.2%)	38.4x	10,306	22,439	17.9	55.4

*Note:* Approximate sequencing depth was calculated relative to the final 1.25‐Gb *P. arminjoniana* assembly and is therefore reported as a *post hoc* descriptive measure of library yield. Metrics were generated using NanoPlot prior to the final read processing steps.

### 2.4 Basecalling and Assembly Preparation

Each of the libraries was processed separately before being concatenated into a single FASTQ file for the assembly and downstream analyses. Basecalling was done using Dorado v1.3.0 in super‐accurate (SUP) mode (min‐qscore 10) via the run‐time ONT software MinKNOW. Adapters were trimmed using Porechop_ABI v0.5.0 (flag ‐abi) (Bonenfant et al. 2023), and the reads were filtered using Chopper v.0.8.0 (‐‐tailcrop 100 ‐‐headcrop 100 ‐‐minlength 90) (De Coster and Rademakers 2023).

### 2.5 Assembly and Scaffolding

The assembly and downstream analyses were all run on the Galaxy EU instance (Galaxy 2026). We did this because the system is user‐friendly, and it is easy to share and reproduce analysis pipelines. We followed the workflow recently used for the assembly and annotation of the Galápagos petrel genome as closely as possible (Sessi et al. 2026), but adapted this to the availability of tools and wrapper options on Galaxy.

We conducted a single *de novo* assembly using Flye v2.9.6 in Nanopore HQ mode and with a single polishing iteration (Kolmogorov 2021; Lin et al. 2016). We polished the resultant assembly once with Medaka v2.1.1 using the model r1041_e82_400bps_sup_v5.0.0 (https://github.com/nanoporetech/medaka).

We checked the assembly for adapters using the NCBI FCS Adapter tool and Eukaryotes taxonomy (https://github.com/ncbi/fcs/wiki/), and screened for contaminants with the NCBI FCS‐GX tool using the NCBI FCS GX database (2023‐01‐24) and taxonomy ID 508676 (https://github.com/ncbi/fcs/wiki/) (Astashyn et al. 2024). To remove mitochondrial contamination, we first identified the mitochondrial contig in the assembly (see Section 2.10). This identified a single mitochondrial contig in the genome assembly that we then removed, providing a cleaned assembly for further analysis.

We purged haplotypic duplications and contig overlaps from the assembly using the purge_dups v1.2.6 pipeline (Guan et al. 2020). We first mapped the reads onto the assembly using minimap2 v2.28 with the *Oxford Nanopore reads to reference mapping* option (Li 2018), then used the Purge_overlaps tool to calculate read depth and cutoff. Next, we split the assembly based on this cutoff, and mapped this split assembly back onto the complete assembly using minimap2. Finally, we used the Purge_overlaps tool to purge haplotypic duplicates and overlaps from the assembly. This process generated a cleaned and purged assembly for scaffolding.

We used the RagTag v2.1.0 tool for reference‐based scaffolding (Alonge et al. 2022). Following Sessi et al. (2026), we used the high‐quality, chromosome‐level assembly for Cory's shearwater as our reference genome (NCBI genome GCA_964195595.2) (Izquierdo Aranega et al. 2024). We specifically used the *hap1* assembly because it includes a full set of sex and autosomal pseudo‐chromosomes. We filled gaps in the scaffolded assembly using TGS‐GapCloser v1.0.3 with the scaffolded assembly and concatenated reads file in FASTA format as input (Xu et al. 2020). The resulting output in FASTA format became our final assembly (*PteroArmin_v1.1*).

Recent WGS studies based on ONT long‐read data in birds suggest assembly quality may be affected by the assembler used (Martin et al. 2026; Sessi et al. 2026). To benchmark our Flye/Medaka assembly, therefore, we also generated an independent *de novo* assembly from the same concatenated ONT read dataset using Hifiasm v0.25.0 in its ONT‐specific assembly mode (Cheng et al. 2021). We specified an estimated genome size of 1.3 Gb and a homozygous coverage of 39x (‐‐hg‐size 1300 m and ‐‐hom‐cov 39) based on the expected genome size and approximate sequencing depth, and used these settings to guide haplotype purging. The Hifiasm assembly was processed through equivalent major downstream stages to the Flye/Medaka assembly, including adapter/contaminant screening, removal of mitochondrial sequence, haplotig purging and RagTag reference‐guided scaffolding against the same Cory's shearwater reference genome. Assembly contiguity and completeness were then assessed using the same SeqKit and BUSCO procedures described below (see Section 2.6) allowing direct comparison with the Flye/Medaka assembly.

### 2.6 Assembly QC and Completeness

We assessed the quality and completeness of our two assemblies (Flye/Medaka and Hifiasm) using the SeqKit_statistics v2.12.0 and BUSCO v.5.8.0 tools (Shen et al. 2016; Simao et al. 2015). For our BUSCO analyses, we used the BUSCO v5 Lineage Datasets (all+2024‐03‐21), DNA mode, and the Metaeuk gene predictor. BUSCO was set‐up to auto‐detect the lineage group to use, which resulted in it selecting the aves_odb10 lineage dataset (creation date: 2024‐01‐08, number of genomes: 62, number of BUSCOs: 8338).

### 2.7 Sex Determination

We determined the sex of our sample using read depth. If read depth of the Z chromosome is approximately equal to the autosomes, then the sample is of a male bird, which has two copies of the Z chromosome. In contrast, if read depth of the Z chromosome is approximately half that of the autosomes, then the sample is of a female bird with only a single copy. To calculate read depth, we mapped our reads onto our scaffolded assembly using minimap2 v2.28. Reads were excluded (*Exclude reads with any of the following flags set*—option) if: (i) a read was unmapped, (ii) the alignment of the read was not primary, (iii) a read failed vendor/platform quality checks, (iv) a read was a PCR or optical duplicate, or (v) an alignment was supplementary. We then calculated read depths using Samtools_coverage v1.22 (Danecek et al. 2021). For autosomes, we summarised the read depth as the median coverage across the 39 scaffolds. Using the Cory's shearwater reference naming, the Z chromosome/scaffold in our assembly was NC_134352.1_RagTag.

### 2.8 Repeat Analysis

We identified repeat regions *de novo* using RepeatModeler v2.0.5 with our final assembly as input (Flynn et al. 2020). This output was then combined with curated vertebrate repeat sequences from the Dfam 3.8 database (https://dfam.org/releases/Dfam_3.8/) to generate a repeats library. We then used this library as input to RepeatMasker v4.1.5 (Smit et al. 2015), which generated a soft‐masked version of our final assembly for annotation.

### 2.9 Genome Annotation

We used Helixer v0.3.6 for the structural annotation of the soft‐masked assembly. Helixer is a gene calling tool based on deep neural networks (DNNs) (Holst et al. 2023; Stiehler et al. 2021). We used the built‐in vertebrate lineage database. We generated summary statistics of the resulting annotation using Genome_annotation_statistics v0.8.4 (Haibao et al. 2015). We then used gffread v2.2.1.4 to generate a protein FASTA from the GFF3 output produced by Helixer (Trapnell et al. 2010), and with our soft‐masked assembly as the reference genome. We assessed the quality and completeness of our protein sequence using BUSCO v5.8.0 and OMArk v0.3.1 (Nevers et al. 2022). BUSCO was set‐up in protein mode, but with all other settings the same as used for assessing assemblies (see Section 2.6). For OMArk, we used the OMAmer database LUCA v2.0.0.

We functionally annotated our protein sequences using a hierarchical evidence framework. Protein sequences were first compared against the UniProtKB Swiss‐Prot (2023_03) reference database using Diamond v2.1.22 (Buchfink et al. 2021). Searches used the BLOSUM62 scoring matrix, Tantan masking algorithm, and an e‐value cutoff of 1e‐10. Only the top‐scoring alignment was retained for each query sequence. We did not impose additional minimum identity or query coverage thresholds at this stage because Swiss‐Prot entries are manually curated and the subsequent hierarchical annotation framework provided additional filtering and validation.

For sequences lacking Swiss‐Prot hits, we used orthology‐based functional annotation from eggNOG_Mapper v2.1.13 with the eggNOG database v5.0.2 (Huerta‐Cepas et al. 2017; Huerta‐Cepas et al. 2016). We opted to seed orthologs computed with Diamond, used BLOSUM62 for the scoring matrix, set the gap costs to *Existence: 11 Extension 1*, set Diamond's sensitivity mode to *sensitive*, and used only non‐electronically curated terms. Lastly, InterProScan v5.59‐91.0 with the InterProScan 5.59‐91 database was employed to identify conserved protein domains and assign Gene Ontology (GO) terms. We opted to run all the applications available (Jones et al. 2014). Where multiple sources provided annotations, priority was given to Swiss‐Prot, followed by eggNOG and InterPro, reflecting decreasing levels of curation and specificity. Proteins lacking support from all methods were annotated as hypothetical proteins.

We ran a final gap‐filling search on proteins lacking annotation using Diamond v2.1.22 with the TrEMBL database. TrEMBL‐derived annotations were considered low confidence unless they met conservative filtering criteria of an *e* value < 1e^–10^ and substantial (50%) query coverage, and unless the matched protein description was biologically informative rather than generic or hypothetical. Only 11 additional proteins met these criteria, and these annotations were not incorporated into the final annotation because they contributed little additional confidence or biological specificity.

We generated a tabular file containing the functional annotation details from these multiple searches and merged this with the Helixer GFF3 output file using a bespoke Python script run in the interactive tool JupyterLab Notebook on Galaxy EU. This generated a final functional (protein) annotation GFF3 file.

We also wanted to include non‐coding RNAs in our functional annotation. To do this, transfer RNA genes were identified using tRNAscan‐SE v2.0 (Chan et al. 2021), and ribosomal RNA genes were predicted using barrnap v0.8 (Seeman 2013). These annotations were incorporated into the final GFF3 as independent features alongside protein‐coding gene models.

### 2.10 Mitochondrial Contig Identification and Removal

To identify mitochondrial sequence for removal from the nuclear genome assembly, we analysed the assembled Flye contigs using MitoHiFi v3.2.3 (Uliano‐Silva et al. 2023). MitoHiFi was used here primarily as a mitochondrial‐contig identification tool rather than to generate a fully curated mitochondrial genome. The pipeline's reference‐search procedure identified the complete mitochondrial genome of *Macronectes giganteus* (GenBank OR731193) as the closest suitable reference, which was then used to identify the mitochondrial contig within the Flye assembly. This contig was removed before haplotig purging, reference‐guided scaffolding and subsequent nuclear‐genome analyses.

As an additional robustness check, we applied the same MitoHiFi procedure to the independently generated Hifiasm assembly. This identified a highly similar mitochondrial contig, allowing us to assess whether recovery of the mitochondrial sequence was sensitive to the whole‐genome assembler used. We compared the Flye‐ and Hifiasm‐derived mitochondrial contigs using minimap2 v2.28 in assembly‐to‐assembly mode.

### 2.11 Comparative Analysis

To place the new *P. arminjoniana* genome within a broader *Pterodroma* comparative context, we analysed our assembly together with eight genomes provided by the Birds 10 K (B10K) project and the published *P. phaeopygia* genome. The B10K genomes comprised *P. alba* (B10K‐D‐PPPC‐3), *P. atrata* (B10K‐D‐PPPC‐5), two *P. heraldica* genomes (B10K‐D‐Raine‐1 and B10K‐D‐PPPC‐7), two *P. arminjoniana* genomes (B10K‐D‐FURG‐8 and B10K‐D‐FURG‐7), *P. neglecta* (B10K‐D‐PPPC‐10), and *P. ultima* (B10K‐D‐PPPC‐2). The two B10K *P. arminjoniana* genomes were labelled *P_arminjoniana_1* and *P_arminjoniana_2*, respectively.

We used BUSCO v5.8.0 with the aves_odb10 lineage dataset to identify single‐copy orthologues recovered across all ten genomes. BUSCO sequences shared by every genome were extracted and grouped by BUSCO gene ID, producing one orthologous sequence set per locus. Each locus was aligned independently using MAFFT v7.526 with the FFT‐NS‐2 algorithm (Katoh et al. 2002; Katoh and Standley 2013). We then used AMAS v1.0 to summarise each alignment and quantify the number of variable and parsimony‐informative sites (Borowiec 2016).

Since BUSCO loci are relatively conserved and may differ substantially in phylogenetic information at this taxonomic scale, we assessed the sensitivity of species‐tree inference to locus informativeness. We generated three BUSCO datasets comprising loci containing at least 1, at least 10, and at least 20 parsimony‐informative sites, respectively. These thresholds retained progressively smaller but more information‐rich sets of loci for species‐tree estimation.

For each retained BUSCO alignment, we estimated an independent maximum‐likelihood gene tree using IQ‐TREE v2.4.0 (Nguyen et al. 2015). Model selection was performed separately for each locus using ModelFinder with the MFP option, and branch support was assessed using 1000 ultrafast bootstrap replicates and 1000 SH‐aLRT replicates. The maximum‐likelihood tree from each locus was retained for downstream species‐tree analysis.

To assess sensitivity to poorly resolved gene‐tree relationships, we conducted each species‐tree analysis using both the original gene trees and a conservatively contracted version of the same gene trees. Internal branches with ultrafast bootstrap support ≤ 10 were collapsed, as were unlabelled internal branches with branch lengths ≤ 1 × 10^−6^, using a bespoke Python script. This reduced the influence of weakly resolved or effectively zero‐length branches while retaining supported relationships.

Species trees were inferred from the collections of individual BUSCO gene trees using ASTRAL‐III under the multispecies coalescent framework (Zhang et al. 2018). ASTRAL was run separately for each of the three locus‐information thresholds, using both uncontracted and contracted gene‐tree sets, giving six species‐tree analyses in total. Branch support on the ASTRAL trees was evaluated using local posterior probabilities. We drew unrooted trees from the ASTRAL output using Newick Display v1.6 (Junier and Zdobnov 2010). We compared topology and support across these six analyses to identify relationships that were robust to both locus information content and gene‐tree uncertainty. We present the contracted trees in the main manuscript, while the corresponding uncontracted trees are available in the online archive (see Data Availability Statement for details).

## 3 Results

### 3.1 Basecalling and Assembly Preparation

The three libraries collectively generated approximately 57.0 Gbp of sequence before final filtering. Following adapter trimming and read filtering, the concatenated dataset used for assembly comprised 55.27 Gbp, equivalent to approximately 44.2x coverage relative to the final 1.25‐Gb assembly. Mean read length was 8565.6 bp, read N50 was 20,907 bp, and 63.5% of reads had a mean quality score greater than Q20.

### 3.2 Assembly and Scaffolding

Assembly quality and completeness remained consistently high across the assembly pipeline, while contiguity improved substantially after purging and reference‐guided scaffolding (Table 2a; Figure 2). Cleaning had little effect on overall assembly size or contiguity, producing a similarly sized assembly of 1.26 Gbp with the same N50 and maximum sequence length. Purging reduced the number of sequences from 2024 to 1383 and decreased the total assembly length from 1.26 Gbp to 1.24 Gbp. This step increased the mean sequence length from approximately 623 kbp to 900 kbp, while maintaining the same N50 of 20.21 Mbp. GC content changed only slightly from 42.99% to 42.84%.

**TABLE 2:** Changes in assembly quality through the genome analysis pipeline for (a) the Flye/Medaka reference assembly and (b) the Hifiasm benchmarking assembly.

(a) Flye assembly				
Metric	Assembly			
	Contig^a^	Cleaned^b^	Purged^c^	Scaffolded^d^
Number of sequences (contigs/scaffolds)	2025	2024	1383	1103
Total length	1.26 Gbp	1.26 Gbp	1.24 Gbp	1.25 Gbp
Mean length	622,437 bp	622,734 bp	900,204 bp	1,130,004 bp
Max. length	65.98 Mbp	65.98 Mbp	65.98 Mbp	220.05 Mbp
N50	20.21 Mbp	20.21 Mbp	20.21 Mbp	85.52 Mbp
L50	21	21	21	5
GC content (%)	42.99%	42.99%	42.84%	42.82%
BUSCO counts (n: 8338)^e^	C: 8136; D: 23; F: 33; M: 169	C: 8136; D: 23; F: 33; M: 169	C: 8136; D: 23; F: 33; M: 169	C: 8139; D: 24; F: 35; M: 164
BUSCO percentages (n: 8338)^e^	C: 97.6%; D: 0.3%; F: 0.4%; M: 2.0%	C: 97.6%; D: 0.3%; F: 0.4%; M: 2.0%	C: 97.6%; D: 0.3%; F: 0.4%; M: 2.0%	C: 97.6%; D: 0.3%; F: 0.4%; M: 2.0%

(b) Hifiasm benchmarking assembly				
Metric	Assembly			
	Contig^a^	Cleaned^b^	Purged^c^	Scaffolded^d^
Number of sequences (contigs/scaffolds)	393	392	350	229
Total length	1.37 Gbp	1.37 Gbp	1.35 Gbp	1.35 Gbp
Mean length	3,491,581 bp	3,500,344 bp	3,867,159 bp	5,911,216 bp
Max. length	115.81 Mbp	115.81 Mbp	115.81 Mbp	222.67 Mbp
N50	26.17 Mbp	26.17 Mbp	26.17 Mbp	88.65 Mbp
L50	15	15	15	5
GC content (%)	43.67%	43.67%	43.55%	43.55%
BUSCO counts (n: 8338)^e^	C: 8134; D: 166; F: 33; M: 171	C: 8134; D: 166; F: 33; M: 171	C: 8132; D: 159; F: 33; M: 173	C: 8133; D: 135; F: 36; M: 169
BUSCO percentages (n: 8338)^e^	C: 97.6%; D: 2.0%; F: 0.4%; M: 2.1%	C: 97.6%; D: 2.0%; F: 0.4%; M: 2.1%	C: 97.5%; D: 1.9%; F: 0.4%; M: 2.1%	C: 97.5%; D: 1.6%; F: 0.4%; M: 2.0%

^a^ Contig assembly.

^b^ Assembly after adapter/contaminant screening and removal of mitochondrial sequence.

^c^ Assembly after haplotig purging.

^d^ Reference‐guided scaffolded assembly.

^e^ BUSCO analyses used the aves_odb10 lineage dataset (*n* = 8338).

**FIGURE 2:**
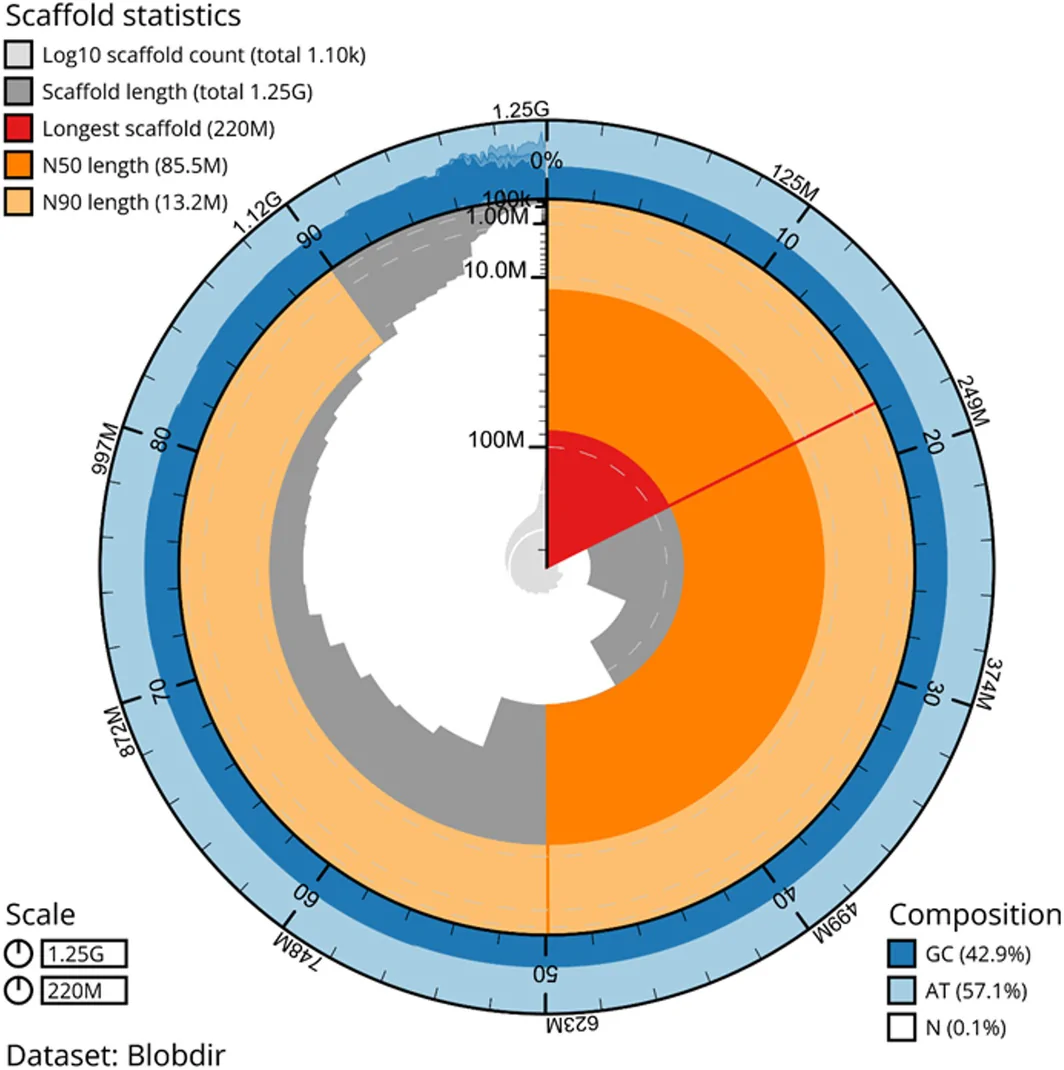
Scaffold‐level summary of the final *P. arminjoniana* genome assembly. Snail plot showing the size distribution and nucleotide composition of the final scaffolded assembly. The assembly spans 1.25 Gb across 1.10 k scaffolds, with the cumulative scaffold length shown around the outer axis. The grey radial histogram represents scaffold length distribution, and the pale outer ring shows scaffold count on a log10 scale. The longest scaffold is 220 Mb, indicated in red. Assembly contiguity metrics are shown as radial sectors, with the N50 scaffold length of 85.5 Mb in orange and the N90 scaffold length of 13.2 Mb in pale orange. The composition ring indicates GC content of 42.9%, AT content of 57.1%, and ambiguous bases of 0.1%.

The largest improvement in assembly contiguity occurred during reference‐guided scaffolding with the mean sequence length increasing to 1.13 Mbp, and the maximum scaffold length rising more than threefold (65.98–220.05 Mbp) (Table 2a). The N50 increased more than fourfold (20.21–85.52 Mbp) and the L50 was reduced from 21 to 5, indicating that half of the assembly length was contained in only five scaffolds. The final scaffolded assembly comprised 1.246 Gb of sequence distributed across 1103 scaffolds, with gaps representing only 0.069% of the total assembly length. SeqKit estimated the genome‐wide GC content at 42.8%, consistent with the snail plot estimate of ~42.9% GC (Table 2a; Figure 2).

Genome completeness remained stable throughout the assembly pipeline (Table 2a). BUSCO analysis consistently recovered 97.6% complete avian BUSCOs with fragmented and missing BUSCOs remaining low (0.4% and 2.0%, respectively). The final assembly using scaffolding recovered 8139 complete avian BUSCOs out of the 8338 expected, slightly higher than in earlier assemblies (8136), while the number of missing BUSCOs decreased from 169 to 164. Using the aves_odb10 lineage dataset, 97.3% of BUSCOs were single‐copy and 0.3% duplicated, indicating a highly complete avian genome assembly with 8115 single‐copy orthologues.

Read depth analysis against the scaffolded assembly showed similar coverage for the autosomes and Z chromosome, with median autosomal coverage of 40.53x and Z chromosome coverage of 43.24x, consistent with the sequenced individual being male (ZZ).

The results of the Hifiasm benchmarking assembly are shown in Table 2b. At contig level, Hifiasm produced substantially fewer sequences than Flye/Medaka (393 versus 2025) and greater contiguity, with an N50 of 26.17 Mbp compared with 20.21 Mbp. BUSCO completeness was effectively identical at this stage (97.6% for both assemblies), although duplicated BUSCOs were higher in the Hifiasm assembly (2.0% vs. 0.3%). Following cleaning, purging and reference‐guided scaffolding, the difference in contiguity was modest: the Hifiasm assembly comprised 229 scaffolds with an N50 of 88.65 Mbp and L50 of 5, compared with 1103 scaffolds, an N50 of 85.52 Mbp and L50 of 5 for the Flye‐based reference. BUSCO completeness remained very similar (97.5% vs. 97.6%), while duplicated BUSCOs remained higher in the Hifiasm assembly (1.6% vs. 0.3%). Thus, Hifiasm provided a modest gain in contiguity but no overall improvement in completeness, supporting the use of the Flye/Medaka assembly as the reference for downstream analyses.

### 3.3 Repeat Analysis

The repeats analysis resulted in 11.55% of the genome being soft‐masked. LINEs dominated the repeat composition (4.4%), most of which was L2/CR1/Rex (4.3%). LTRs were 1.83%, DINA transposons 0.41% and SINEs 0.21%. Unclassified repeats represented 3.58%.

### 3.4 Genome Annotation

The Helixer structural annotation produced one transcript model per gene (*n* = 18,099 genes). Most genes were multi‐exonic, with 17,619 genes, 97.3% containing more than one exon, and the annotation included 232,143 distinct exons with a mean of 12.8 exons per gene. Annotated genic regions covered 53% of the genome, comprising approximately 60 Mb of exonic sequence and 604 Mb of intronic sequence. Exons had a higher GC content (49%) than genes overall (43%) and introns (42%).

The Helixer protein annotation was highly complete and taxonomically consistent. BUSCO analysis against aves_odb10 recovered 93.6% complete BUSCOs with low duplication (0.4%) and 4.3% missing BUSCOs. OMArk analysis against Neognathae conserved HOGs gave a comparable completeness estimate with 94.45% single‐copy, 3.13% duplicated, and 2.42% missing HOGs. Most proteins were assigned to lineage‐consistent gene families (92.1%), and no contaminants were detected.

Functional annotation assigned putative or domain‐supported annotations to 17,175 of 18,099 protein‐coding transcripts (94.9%). Most annotations were supported by curated Swiss‐Prot homology (16,475; 91.0%), while a smaller number were assigned using orthology‐ or domain‐based evidence (eggNOG: 690, 3.8%; InterPro: 10, respectively). The remaining 972 transcripts (5.4%) lacked functional support and were annotated as hypothetical proteins. The final GFF3 also included 399 non‐coding RNA features consisting of 333 tRNAs and 66 rRNAs.

### 3.5 Mitochondrial Contig Identification and Removal

MitoHiFi identified a 20,278‐bp mitochondrial contig within the Flye assembly. Applying the same procedure to the independently generated Hifiasm assembly recovered a highly similar mitochondrial contig of 21,385 bp. Assembly‐to‐assembly alignment with minimap2 spanned 99.96% of both contigs, with an estimated sequence divergence of only 0.24%, indicating that the unusual mitochondrial sequence was recovered consistently by both whole‐genome assemblers. The Hifiasm‐derived contig was 1107 bp longer than the Flye‐derived contig, showing that the precise length and architecture of the expanded mitochondrial region were not fully resolved.

### 3.6 Comparative Analysis

The genomes included in the comparative analysis varied substantially in assembly contiguity, although most had high BUSCO completeness (Table 3). The new *P. arminjoniana* reference was more contiguous than the two B10K *P. arminjoniana* assemblies, with a scaffold N50 of 85.52 Mb compared with 18 and 17 Mb, respectively, while BUSCO completeness was very similar among the three genomes (97.6%, 97.3% and 97.4%). Across all ten genomes, 6191 single‐copy BUSCO loci were recovered and retained for phylogenomic analysis.

**TABLE 3:** Comparative assembly metrics and sequencing technologies for the *Pterodroma* genomes used in the comparative analyses.

English name	Scientific name	Genome ID/Source	Genome label (our study)	Contig N50 (Mb)	Number of contigs	Scaffold N50 (Mb)	Number of scaffolds	Assembly size (Gbp)	BUSCO completeness (%)	Sequencing platform
Trindade petrel	*P. arminjoniana*	New reference genome	P_arminjoniana	20.21	2025	85.52	1103	1.250	97.6	ONT PromethION 2 Solo
Phoenix petrel	*P. alba*	B10K‐D‐PPPC‐3	P_alba	0.157	47,523	0.494	39,285	1.241	95.2	MGI stLFR (100‐bp paired‐end)
Henderson petrel	*P. atrata*	B10K‐D‐PPPC‐5	P_atrata	0.164	43,142	0.517	34,613	1.204	95.6	MGI stLFR (100‐bp paired‐end)
Herald petrel	*P. heraldica*	B10K‐D‐Raine‐1	P_heraldica_1	0.240	30,194	13	20,378	1.215	97.3	MGI stLFR (100‐bp paired‐end)
Trindade petrel	*P. arminjoniana*	B10K‐D‐FURG‐8	P_arminjoniana_1	0.267	33,717	18	24,870	1.239	97.3	MGI stLFR (100‐bp paired‐end)
Trindade petrel	*P. arminjoniana*	B10K‐D‐FURG‐7	P_arminjoniana_2	0.248	33,357	17	23,652	1.233	97.4	MGI stLFR (100‐bp paired‐end)
Herald petrel	*P. heraldica*	B10K‐D‐PPPC‐7	P_heraldica_2	0.277	32,678	3	24,410	1.211	97.0	MGI stLFR (100‐bp paired‐end)
Kermadec petrel	*P. neglecta*	B10K‐D‐PPPC‐10	P_neglecta	0.047	125,896	0.049	121,592	1.204	84.0	MGI stLFR (100‐bp paired‐end)
Galápagos petrel	*P. phaeopygia*	GAPE_003 (BioProject PRJNA1275046; BioSample SAMN49010279)	P_phaeopygia	46	57	74	45	1.346	97.5	ONT PromethION 2 Solo
Murphy's petrel	*P. ultima*	B10K‐D‐PPPC‐2	P_ultima	0.162	50,834	0.506	42,868	1.204	95.4	MGI stLFR (100‐bp paired‐end)

*Note:* The new Trindade petrel reference genome is shaded grey. For the new *P. arminjoniana* reference, contig metrics refer to the Flye/Medaka de novo assembly and scaffold metrics to the final reference‐guided assembly (see Table 2a). B10K genomes were generated using MGI single‐tube Long Fragment Read (stLFR) sequencing with 100‐bp paired‐end reads (Oksnebjerg et al. 2026).

The amount of phylogenetic information varied substantially among BUSCO loci. Of the 6191 shared loci, 5556 contained at least one parsimony‐informative (PI) site, 1874 contained at least 10 PI sites, and 1260 contained at least 20 PI sites. We therefore inferred ASTRAL species trees across these three increasingly informative locus subsets. The contracted species trees showed broadly similar major relationships across all three thresholds (Figure 3).

**FIGURE 3:**
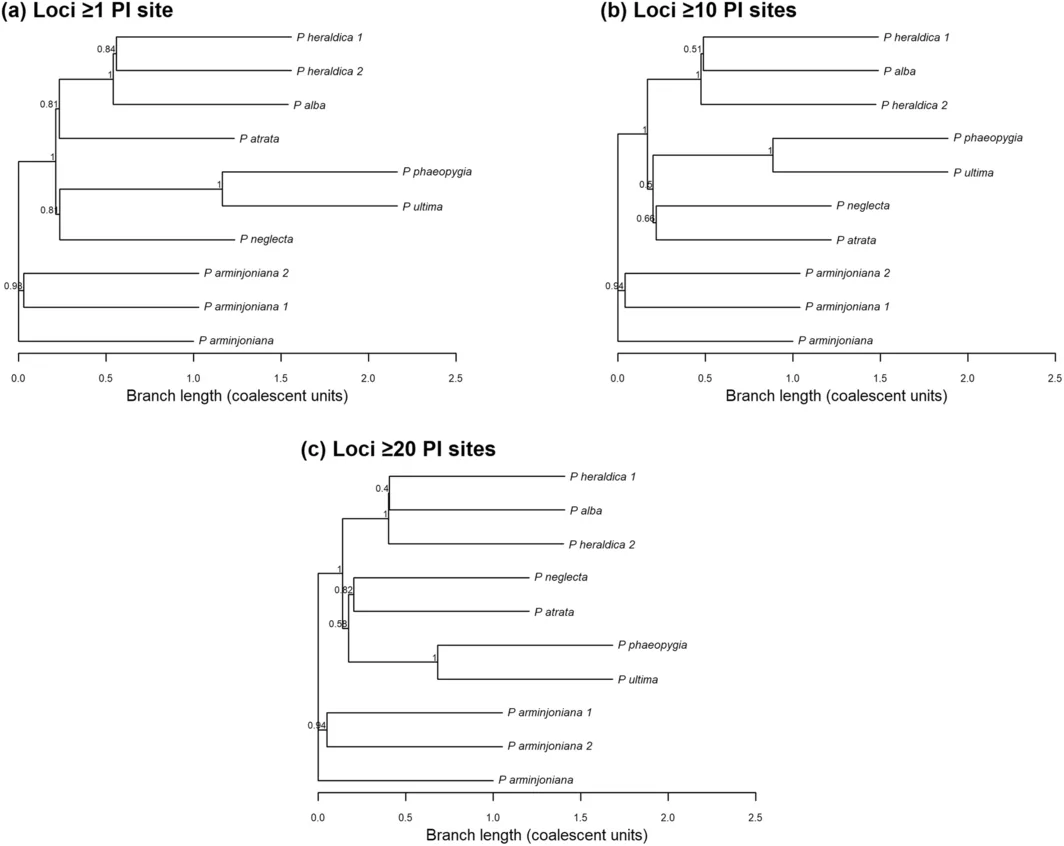
ASTRAL‐III species trees inferred from contracted BUSCO gene trees across three locus‐informativeness thresholds. Species trees were inferred using BUSCO loci containing (a) at least 1, (b) at least 10, or (c) at least 20 parsimony‐informative (PI) sites. Prior to ASTRAL analysis, very poorly supported or effectively zero‐length internal branches in individual gene trees were contracted. Values at internal nodes are ASTRAL local posterior probabilities. Trees are shown unrooted, and branch lengths are plotted on the scales shown for each panel. A scale in coalescent units is shown on each plot.

The two B10K *P. arminjoniana* genomes consistently formed a strongly supported pair in all three contracted analyses, with local posterior probabilities of 0.98, 0.94 and 0.94 for the ≥ 1, ≥ 10 and ≥ 20 PI‐site datasets, respectively (Figure 3). However, the new *P. arminjoniana* reference remained outside this pair in each analysis, such that the three genomes labelled as *P. arminjoniana* did not form an exclusive monophyletic group. The relationship between *P. ultima* and *P. phaeopygia* was also recovered with very high support across all three analyses. Some relationships elsewhere in the tree were less stable. In particular, the relative placement of *P. atrata* and *P. neglecta* varied among analyses: they were not recovered as a pair in the ≥ 1 PI‐site tree, but were grouped together in the ≥ 10 and ≥ 20 PI‐site analyses, with local posterior probabilities of 0.66 and 0.82, respectively. Relationships within the *P. alba* / *P. heraldica* part of the tree were also less strongly supported in the more stringently filtered datasets.

## 4 Discussion

We present a highly contiguous and complete long‐read reference genome for the Trindade petrel *Pterodroma arminjoniana* , a wide‐ranging procellariiform seabird of conservation and eco‐evolutionary importance. Using Oxford Nanopore long‐read sequencing, we generated a 1.25 Gb genome assembly from a male bird with high contiguity, low unresolved sequence content, and strong completeness. The final scaffolded assembly comprised 1103 scaffolds, had a scaffold N50 of 85.5 Mb, and recovered 97.6% of expected avian BUSCOs. We also produced a structural and functional annotation comprising 18,099 protein‐coding genes, most of which could be assigned functional support, and placed the new assembly in a broader *Pterodroma* phylogenomic context. Together, these resources provide a reference‐quality genome for future studies of taxonomy and systematics, morphology, hybridisation, gene flow, population structure, conservation genomics, and comparative genomics in *P. arminjoniana* and related gadfly (*Pterodroma*) petrels.

The final Flye/Medaka assembly represents a substantial improvement in contiguity over the initial *de novo* assembly while maintaining consistently high completeness (Table 2). Cleaning had little effect on assembly size or BUSCO recovery, whereas purging reduced the number of sequences and total assembly length, consistent with the removal of redundant or haplotypic sequence. The largest improvement followed reference‐guided scaffolding and gap filling, which increased scaffold N50 to 85.52 Mb while retaining 97.6% BUSCO completeness. We generated a second assembly from the same ONT read dataset using Hifiasm because recent studies have shown variation in assembler performance between ONT datasets (Martin et al. 2026; Sessi et al. 2026). Hifiasm produced a more contiguous contig assembly and substantially fewer sequences overall, but after equivalent cleaning, purging and scaffolding the difference in scaffold‐level contiguity was modest (N50 = 88.65 Mb versus 85.52 Mb; L50 = 5 for both), while BUSCO completeness was essentially identical (97.5% vs. 97.6%) although duplicated BUSCOs remained higher in the Hifiasm assembly (1.6% vs. 0.3%). Thus, the benchmark did not reveal evidence that the Flye/Medaka assembly was materially incomplete or suboptimal, and instead showed that both approaches produced highly complete and contiguous assemblies with different trade‐offs between contiguity and residual duplication. We selected the Flye/Medaka assembly as the primary reference for downstream annotation, read mapping and comparative genomic analyses because its lower proportion of duplicated BUSCOs provides a more conservative representation of single‐copy loci, reducing the risk of paralogy‐driven artefacts in ortholog extraction. However, because chromosome‐scale organisation was generated through reference‐guided scaffolding, scaffold order and orientation should still be interpreted with caution. Independent long‐range data, such as Hi‐C, would be required to validate local scaffold structure and lineage‐specific rearrangements.

The repeat and annotation analyses further support the quality and utility of the genome assembly. Repetitive sequence accounted for 11.55% of the genome, which is broadly consistent with expectations for an avian genome (Benham et al. 2024; Bravo et al. 2021). Following soft‐masking, Helixer predicted 18,099 protein‐coding genes, most of which were multi‐exonic, and the resulting protein set showed high completeness and taxonomic consistency. BUSCO recovered 93.6% complete avian orthologues from the predicted proteins, while OMArk assigned 92.1% of proteins to lineage‐consistent gene families and detected no contamination. Avian genomes typically consist of 15,000–23,000 genes (Zhang et al. 2014; Botero‐Castro et al. 2017). Together with the low duplication and contamination signals from BUSCO and OMArk, this suggests that the annotation is neither substantially depleted nor obviously inflated by redundant or contaminant gene models. Functional annotation was also extensive with 94.9% of protein‐coding transcripts receiving support from Swiss‐Prot, eggNOG, or InterPro, and the final GFF3 additionally incorporating predicted tRNAs and rRNAs. These results indicate that the annotation is suitable for broad comparative, population genomic, and functional studies, although the absence of species‐specific transcriptome data means that isoform diversity and some difficult‐to‐predict genes will require further analysis.

MitoHiFi identified an unusually long mitochondrial contig in the *P. arminjoniana* assembly. The primary purpose of this analysis was to identify mitochondrial sequence for removal from the nuclear genome assembly, and this objective was achieved consistently in both the Flye and independently generated Hifiasm assemblies. The two mitochondrial contigs were highly concordant in sequence, although they differed modestly in total length, indicating that the precise architecture of the expanded region is not fully resolved. The unusually large size is consistent with the possibility of duplicated or repetitive mitochondrial sequence, as has been reported in other Procellariiformes (Torres et al. 2019). We therefore regard the mitochondrial contig as robustly identified for the purposes of our nuclear‐genome assembly, while avoiding detailed interpretation of its internal structure. Resolving the organisation, copy number and annotation of the expanded region will require a dedicated mitogenomic analysis and is beyond the scope of the present genome‐resource study.

Comparative analyses place the new *P. arminjoniana* assembly within a growing but still limited set of genomic resources for gadfly petrels and other procellariiform seabirds (Sessi et al. 2026; Foote et al. 2026; Pan et al. 2019; Cuevas‐Caballe et al. 2022; Huynh et al. 2023) (see also Table 3). Our assembly is similar in overall size to the other *Pterodroma* genomes but is substantially more contiguous than the two existing B10K *P. arminjoniana* assemblies, while all three show similarly high BUSCO completeness. The multispecies‐coalescent analyses placed the new reference genome within the wider *Pterodroma* radiation, but the three genomes labelled as *P. arminjoniana* did not form an exclusive monophyletic group. Instead, the two B10K *P. arminjoniana* genomes consistently formed a strongly supported pair across the contracted ASTRAL trees (Figure 3), while the new reference remained outside this pair.

This pattern is potentially consistent with the known complexity of species limits, secondary contact on particular islands (e.g., Round Island [Indian Ocean] and Motu Nui off Rapa Nui [Pacific Ocean]) and hybridisation within this group (Booth‐Jones et al. 2017; Brown et al. 2011; Brown et al. 2010; Plaza et al. 2023), but it should be treated as hypothesis‐generating rather than as evidence of species‐level paraphyly. Importantly, the result was recovered across analyses using increasingly informative BUSCO subsets and after contraction of poorly resolved gene‐tree branches, indicating that it is not simply a consequence of including large numbers of weakly informative loci. Since the species‐tree analyses were restricted to single‐copy BUSCO loci recovered across all sampled genomes, differences in overall assembly contiguity should have limited direct influence on the retained phylogenomic dataset, although poorer assemblies may have reduced the total number of loci available for analysis. Distinguishing among incomplete lineage sorting, introgression, deep population structure, sample provenance and taxonomic uncertainty will require denser population‐level sampling and genome‐wide tests of admixture.

Relationships elsewhere in the tree were also not uniformly stable. In particular, the relative placement of *P. atrata* and *P. neglecta* varied among the ASTRAL sensitivity analyses, although these taxa were grouped together in the more stringently filtered ≥ 10 and ≥ 20 PI‐site datasets. This instability, together with the comparatively low completeness of the available *P. neglecta* assembly, argues for caution in interpreting this relationship. Nevertheless, previous work has considered *P. atrata* most closely related to *P. heraldica* (Brooke and Rowe 1996; Flood et al. 2022), further illustrating the ongoing taxonomic and systematic uncertainty within the genus.

Our reference genome provides an important platform for future ecological, evolutionary and conservation genomic studies of *P. arminjoniana* and its close relatives. It will facilitate genome‐wide analyses of population structure, demographic history, gene flow and hybridisation across the Trindade petrel complex, including comparisons among island populations, and sympatric and seasonally distinct breeding assemblages. This is particularly important given the extensive gene flow and hybridisation observed across ocean basins in this group (Booth‐Jones et al. 2017; Brown et al. 2011; Brown et al. 2010). The assembly will also improve read mapping and variant discovery in future resequencing studies, supporting investigations of allochronic breeding, local adaptation, introgression and conservation management. More broadly, the annotated genome adds to the growing genomic resources available for *Pterodroma* and other procellariiform seabirds, enabling comparative studies of genome organisation, gene content, sex‐linked variation and the evolution of pelagic seabird life histories.

In conclusion, our *P. arminjoniana* reference genome fills an important gap in genomic resources for gadfly petrels and provides a platform for moving studies of this taxonomically complex group from marker‐based inference towards genome‐wide analysis. This resource will support future work on hybridisation, population structure, conservation and comparative seabird genomics across *Pterodroma* and related Procellariiformes.

## Author Contributions

**Mariana Scain Mazzochi:** data curation (lead), formal analysis (lead), methodology (lead), writing – original draft (lead). **Olga L. Herrera‐Trujillo:** data curation (equal), formal analysis (equal), methodology (equal), writing – review and editing (equal). **Ken Norris:** conceptualization (lead), data curation (lead), formal analysis (lead), writing – original draft (lead). **Malcolm A. C. Nicoll:** resources (supporting), writing – review and editing (supporting). **Paula Plaza:** resources (supporting), writing – review and editing (supporting). **David A. Stewart:** resources (supporting), writing – review and editing (supporting). **Maria P. Dias:** supervision (supporting), writing – review and editing (supporting). **Mónica C. Silva:** supervision (supporting), writing – review and editing (supporting). **Leandro Bugoni:** conceptualization (lead), data curation (equal), formal analysis (equal), funding acquisition (lead), project administration (lead), supervision (lead), writing – review and editing (equal).

## Funding

This work was supported by Coordenação de Aperfeiçoamento de Pessoal de Nível Superior, CAPES Finance Code 001, Conselho Nacional de Desenvolvimento Científico e Tecnológico (CNPq) (310145/2022‐8, 405555/2012‐1, 422759/2016‐3, 557152/2009‐7).

## Conflicts of Interest

The authors declare no conflicts of interest.

## Data Availability

The *Pterodroma arminjoniana* reference genome assembly will be available via GenBank under accession JBYHGU000000000. Raw Oxford Nanopore sequencing reads have been deposited in the NCBI Sequence Read Archive (SRA) and will be accessible under BioProject PRJNA1465706. Key intermediate outputs from the assembly and annotation workflow, including assembly FASTA files, quality assessment outputs, repeat and gene annotation files, comparative analysis files, scripts and workflow documentation are available through Zenodo (https://doi.org/10.5281/zenodo.20442205).
